# Targeting Galectins With Glycomimetics

**DOI:** 10.3389/fchem.2020.00593

**Published:** 2020-08-07

**Authors:** Sara Bertuzzi, Jon I. Quintana, Ana Ardá, Ana Gimeno, Jesús Jiménez-Barbero

**Affiliations:** ^1^CIC bioGUNE, Basque Research Technology Alliance, Derio, Spain; ^2^Ikerbasque, Basque Foundation for Science, Bilbao, Spain; ^3^Department of Organic Chemistry II, Faculty of Science and Technology, University of the Basque Country - UPV-EHU, Leioa, Spain

**Keywords:** galectins, glycomimetics, glycans, molecular recogntion, drug design

## Abstract

Among glycan-binding proteins, galectins, β-galactoside-binding lectins, exhibit relevant biological roles and are implicated in many diseases, such as cancer and inflammation. Their involvement in crucial pathologies makes them interesting targets for drug discovery. In this review, we gather the last approaches toward the specific design of glycomimetics as potential drugs against galectins. Different approaches, either using specific glycomimetic molecules decorated with key functional groups or employing multivalent presentations of lactose and N-acetyl lactosamine analogs, have provided promising results for binding and modulating different galectins. The review highlights the results obtained with these approximations, from the employment of S-glycosyl compounds to peptidomimetics and multivalent glycopolymers, mostly employed to recognize and/or detect *h*Gal-1 and *h*Gal-3.

## Introduction

**Figure 1:**
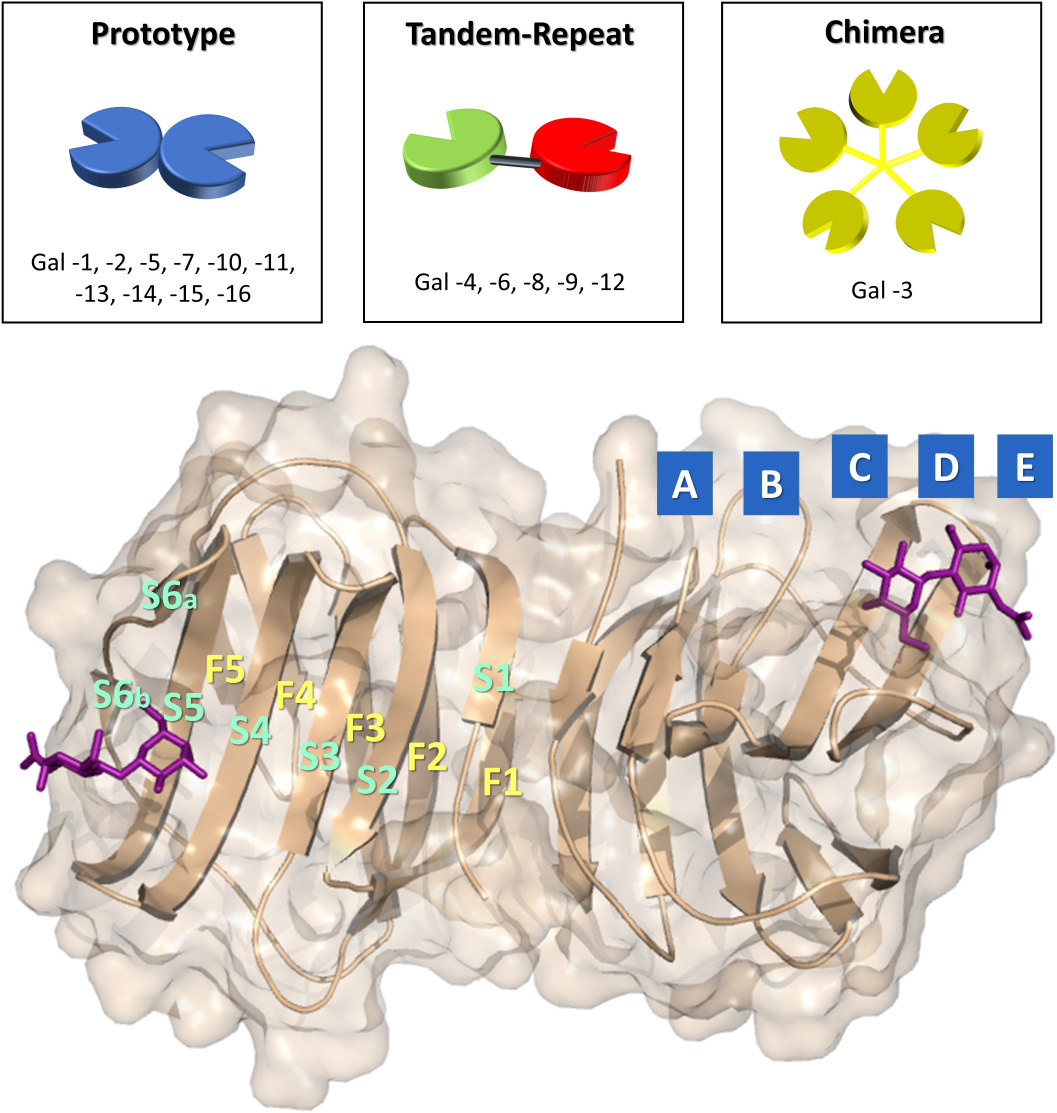
Above: representation of the three group of galectins. On the bottom: representation of the CRD of *h*Gal-1 in complex with N-acetyllactosamine (PDB ID: 1W6P) with subsites (**A, B, C, D**, and **E**) of the binding site and architecture of the beta strands (S1, S2, S3, S4, S5, S6, F1, F2, F3, F4, F5, F6) highlighted.

**Figure 2:**
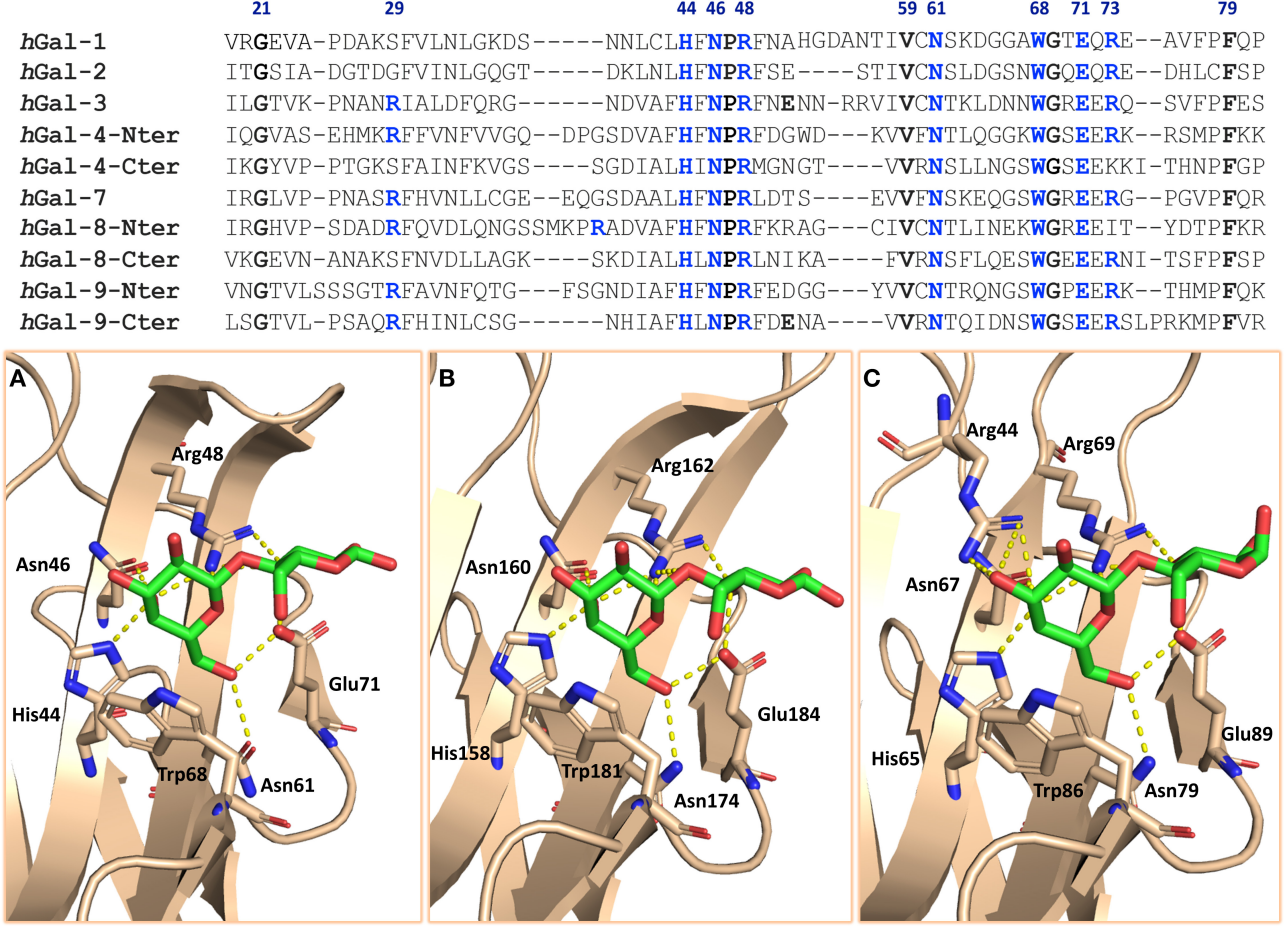
On the top: sequence alignment of the galectins discussed in this review. The residues conserved among galectins are highlighted in bold; the blue ones are those mainly involved in the interaction with lactose. On the bottom: galectin's binding sites of complexes **(A)** *h*Gal-1/lactose (PDB ID: 1GZW), **(B)** *h*Gal-3/lactose (PDB ID: 2NN8), and **(C)** *h*Gal-8-Nterminal/lactose (PDB ID: 5T7S). Residues involved in the interaction are displayed as sticks; H bonds are displayed as yellow dots.

Galectins are a large family of glycan-binding proteins with preference for β-galactoside- (β-Gal) containing structures. In mammals, 16 different members have been described, which can be found both in the intra- and extracellular environments. On the basis of the supramolecular organization of their carbohydrate-recognition domains (CRDs), they are classified (Figure 1) as prototype galectins, when a single CRD spontaneously forms homodimers (human galectins-1, -2, -5, -7, -10, -11, -13, -14, -15, -16), as tandem-repeat galectins, when two homologous CRDs are connected by a short aminoacidic linker (human galectin-4, -6, -8, -9, -12), or as chimera-type galectins (human galectin-3), when the CRD is connected to an N-terminal aminoacidic non-lectin region (Leffler et al., 2002; Yang et al., 2008; Dings et al., 2018). The sequence identity between galectins is moderately high, and the CRD's structure of six (from S1 to S6) and five (from F1 to F5) antiparallel beta strands is always conserved (Figures 1, 2). The β-Gal-binding site is located in the S-face, centered on subsite C and extended away, occupying subsites A, B, D, and E, which display variations between the galectin members (Figure 1).

The canonical binding site of galectins is shallow and solvent exposed, and the interaction with β-galactosides occurs through several hydrogen bonds between the hydroxyl groups of the β-Gal and specific histidine, arginine, and asparagine galectin residues and through the CH-π stacking interaction between the non-polar face of the β-Gal moiety and a conserved tryptophan residue (Figure 2). Even though these residues in the binding site are highly conserved, the divergence in few neighboring sites may modulate the glycan-binding preferences among galectins (Johannes et al., 2018).

Galectins are involved in a wide range of biological activities, such as homeostasis, apoptosis, and vascular embryogenesis (Chan et al., 2018). Their involvement in pathological contexts has been described for inflammation (Brinchmann et al., 2018), host-pathogen interaction (Vasta, 2009), antibacterial autophagy (Weng et al., 2018), and cancer (Liu and Rabinovich, 2005; Girotti et al., 2020). In fact, many members of the family are associated with the phenomena of carcinogenesis being involved in processes such as apoptosis, adhesion, migration, cell transformation, invasion, metastasis, immune escape, and angiogenesis (Dings et al., 2018). The heterogeneous roles of galectins in these physiological and pathophysiological processes have motivated the rational design of fine-tuned inhibitors with precise selectivity and specificity. The characterization at the molecular level of the interaction of galectins with their natural binders has provided the structural basis for the design of potent antagonists (Ardá and Jiménez-Barbero, 2018; Valverde et al., 2019a).

Notwithstanding carbohydrates are the natural ligands of galectins, they are not the best antagonist candidates because of their inherent properties that make them not good enough as pharmacological agents. The main disadvantages are the low metabolic stability and high hydrophilicity. Moreover, it is well-known that the affinities that characterize single carbohydrate-lectin interactions are usually rather weak, in the μM-mM range, far from the nM scale required for a good drug candidate. This evidence derives from many factors intrinsically connected to the nature of carbohydrates, such as the absence of hydrophobic groups that can interact with the protein surface, the hydrogen bond-based interplay with the binding site that suffers from the competition with the bulk water, and the large enthalpic penalties for desolvation of the shallow binding site (Hevey, 2019).

Thus, in the last two decades, molecular scaffolds differently derivatized have been employed as mimetics capable of recognizing and blocking galectins, especially human galectins 1 and 3 (*h*Gal-1 and *h*Gal-3) (Tellez-Sanz et al., 2013; Blanchard et al., 2014, 2016; Girard and Magnani, 2018). These glycomimetics are molecules that mimic carbohydrates structurally and functionally but display improved pharmacological properties (Magnani and Ernst, 2009).

The majority of the molecules synthesized for this purpose are based on sugar scaffolds, although some non-saccharide-based inhibitors, as peptide-based mimetics, have also been reported. The chemical modifications of those based on sugar scaffolds mainly concern the replacement of the endocyclic oxygen of the pyranose ring (carbasugars, iminosugars, thiosugars, and phosphasugars) or the exocyclic oxygen (C-glycosyl, N-glycosyl, S-glycosyl). Indeed, the substitution of the bridging exocyclic oxygen by other atoms has been proved to be effective in increasing the resistance of carbohydrates to hydrolysis (Tamburrini et al., 2019).

The workflow for the development of new glycomimetics aims at finely modulate affinity and stability, as well as selectivity. Since, as discussed, the CRD of mammalian galectins is highly conserved, with minor amino acid sequence variation between members of the family, achieving selective glycomimetics is somewhat difficult and challenging (Figure 2) (Compain, 2018; Tamburrini et al., 2019).

In addition to the modifications of the endocyclic or exocyclic oxygen of the sugar scaffold, which modulate mostly stability and conformation, the introduction of new functional groups as substituents enables reaching new binding pockets and establishes additional contacts, in order to increase affinity and selectivity. Moreover, the development of potent inhibitors usually resorts to the employment of multivalent ligands, which exploits lectin avidity, and allows reaching increased affinities. We have arbitrarily divided the glycomimetics design in this area into two categories: strategies that exploit the presentation of a single strong inhibitor (monovalent carbohydrate-based inhibitors) and those that use multimerized ligands (multivalent carbohydrate-based inhibitors). Below, the most recent and promising examples for both types of inhibitors are presented and contextualized.

## Single Presentation Strategies

As canonical natural ligands of galectins, lactose or N-acetyllactosamine have been the most-used scaffolds to build synthetic glycomimetics, although monosaccharides and other disaccharides have also been taken into consideration and have led to the synthesis of mimetics with promising characteristics (Dings et al., 2018). All the monovalent carbohydrate-based inhibitors can be defined as modified Gal-containing mono- or disaccharides decorated with diverse molecular fragments.

On the molecular basis, the interaction of galectins with β-galactoside sugars, besides the stacking interaction with the conserved Trp moiety, mainly involves hydroxyl O4 and O6 of galactose (Figure 2). Therefore, the other positions are free to accept modifications that can enhance the effectiveness of the mimetic.

In fact, lactosamine derivates with modifications at Gal-C3 (4-phenoxyaryl, via ether or triazole linkages) and Glc-N2 (attachment of an aromatic phthaloyl or benzoyl) have been synthetized, and their affinities have been measured using fluorescence polarization assays (Sörme et al., 2004). These molecules reached dissociation constants in the low μM range for *h*Gal-3 and *h*Gal-1 (Van Hattum et al., 2013). Especially, compound **1** (*K*_*D*_ 1.2 μM) was far more selective toward *h*Gal-3 over *h*Gal-1 (230-fold) (**Figure 5**). The larger aromatic substitution at Glc-N2 is better accommodated in the binding site of *h*Gal-3. Nevertheless, analogous modifications on a non-hydrolysable thio-digalactoside (TDG) scaffold led to the synthesis of glycomimetics showing high affinity (Van Hattum et al., 2013).

**Figure 3:**
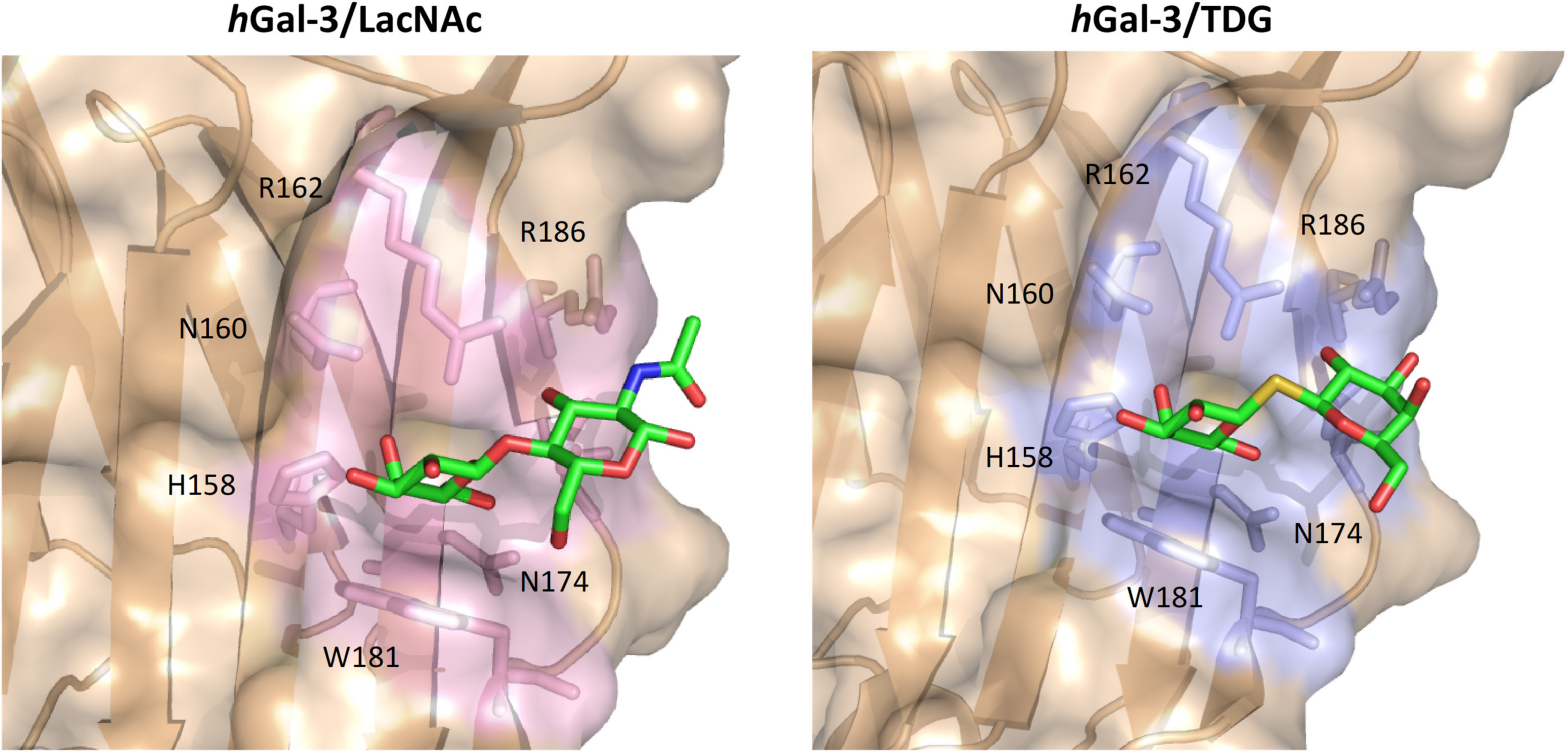
Binding site of the *h*Gal-3/LacNAc (PDB ID: IKJL) and *h*Gal-3/TDG (PDB ID: 4JC1) complexes. Key residues for binding are reported as sticks.

**Figure 4:**
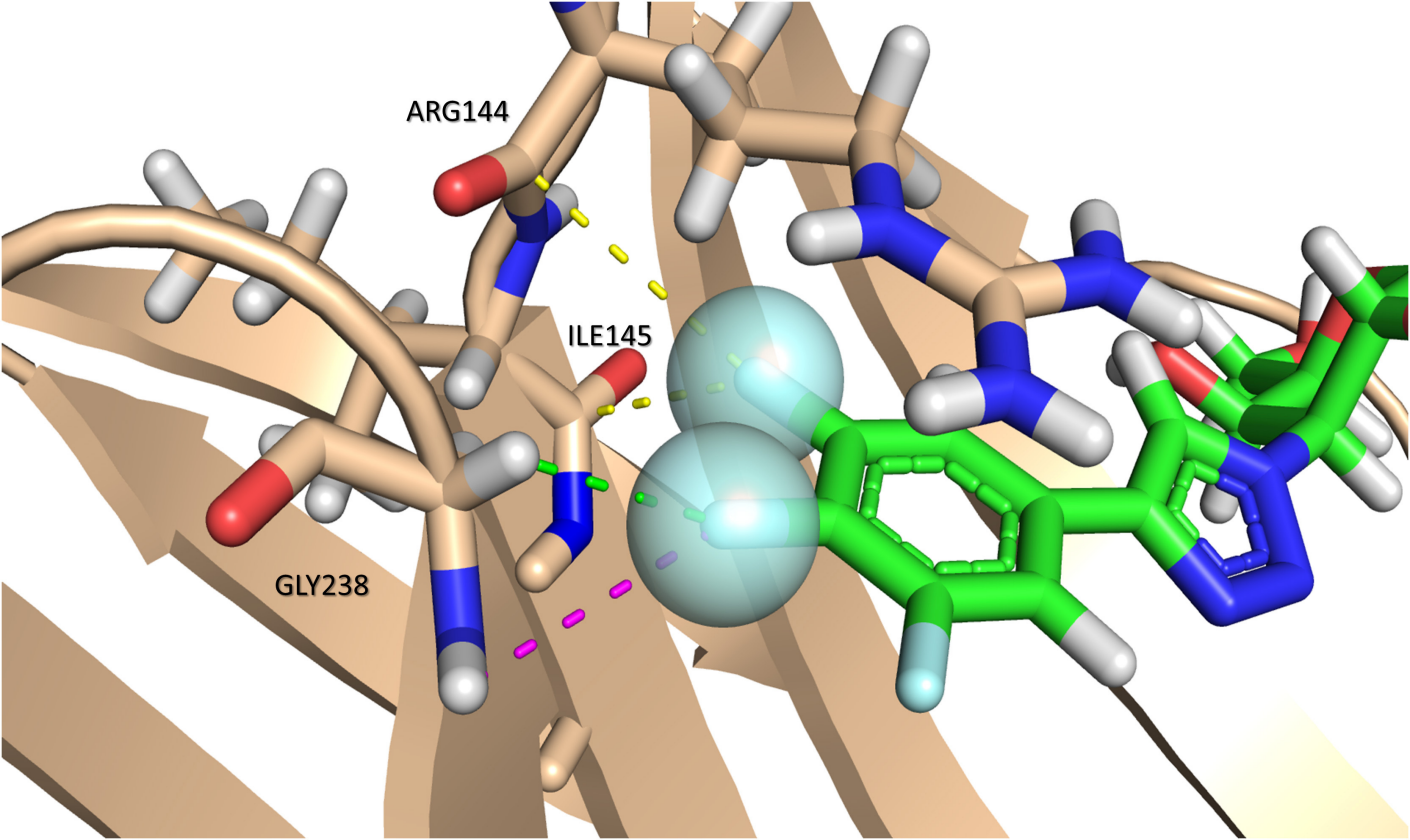
Multipolar interactions between the fluorine atoms (blue sphere) of compound **13** and *h*Gal-3. C-F···H-N interactions are represented with purple dashes. C-F···H-Cα interactions with green dashes. The C-F···C=O interaction with the backbone amides of Arg144 and Ile145 is represented with yellow dashes. PDB ID: 6QLP. The interactions are represented with analog **13** instead of **TD139** since it lacks C-F···H-N and C-F···H-Cα interactions.

The TDG scaffold (Figure 3) has proved to be an effective platform for designing glycomimetics. These molecules reproduce the interactions of lactosides at subsites C and D of galectins with the advantage of being hydrolytically stable (Figures 3, 4).

**Figure 5:**
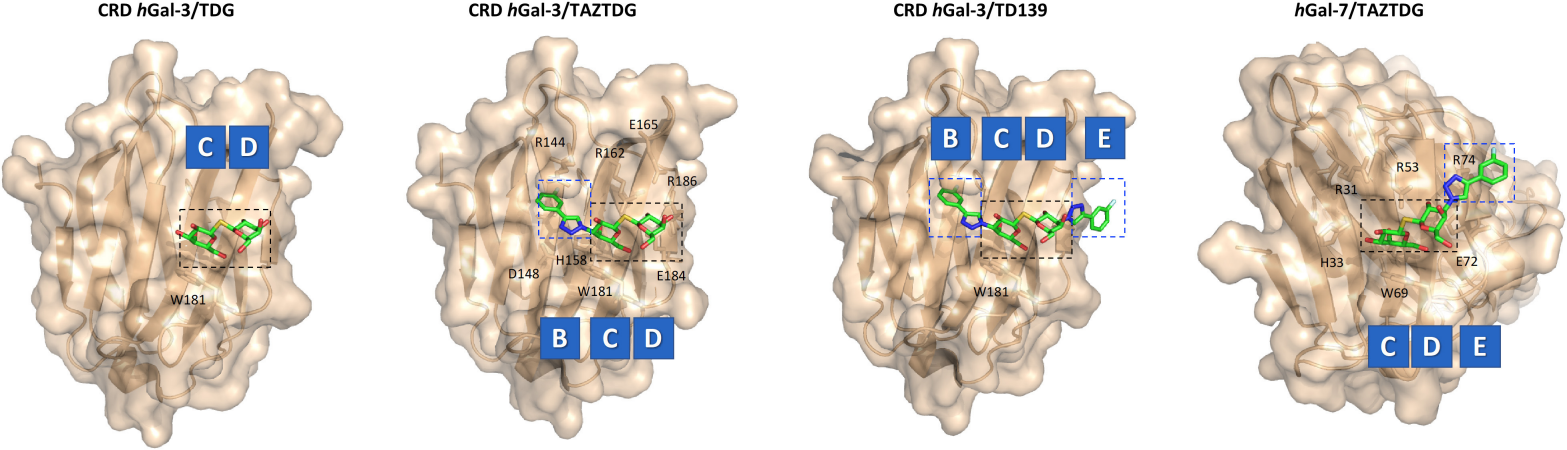
X-ray structures of *h*Gal-3/TDG (PDB ID: 4JC1), *h*Gal-3/TAZTDG (PDB ID: 5H9R), *h*Gal-3/TD139 (PDB ID: 5H9P), and *h*Gal-7/TAZTDG (PDB ID: 5H9S) complexes. Galectin's subsites and the key residues for the interaction are highlighted.

In particular, **TD139**, a symmetric TDG substituted with aromatic moieties at each C3 position, has proved to be a very good antagonist of *h*Gal-1 and *h*Gal-3 (MacKinnon et al., 2012; Figure 5). Furthermore, the compound includes fluorinated substituents, which can have an enormous impact in protein-ligand interactions. Besides decreasing hydrophobicity, the presence of fluorine atoms provides multiple ways to interact with protein moieties, such as multipolar interactions with carbonyl groups, amides, or dipole-dipole interactions (Figure 4).

**TD139** is one of the few examples of galectin's antagonist in advanced clinical trials (Galecto Biotech). In particular, it has been employed as a therapeutic agent against *h*Gal-3 for the treatment of Idiopathic Pulmonary Fibrosis. Since the molecule is not well-absorbed orally and must be given parenterally, the method of administration is inhalation of dry powder.

Hsieh et al. (2016) have taken advantage of this scaffold to target and analyze the interaction with *h*Gal-1, -3, and -7. In order to distinguish the implication of each aromatic group of TD139 in the binding, **TAZTDG**, a mimetic with only one aromatic substitution, was studied (Figure 5). ^15^N-^1^H HSQC NMR titrations, ^19^F relaxation NMR experiments, Isothermal Titration Calorimetry (ITC), and X-ray have unearthed that for *h*Gal-1 and -7, the aromatic ring contributes more to the binding at the subsite E, whereas for *h*Gal-3, the fluorophenyl group is mainly interacting with subsite B (Figure 5). This kind of information can be employed for the rational design of new inhibitors, since different substituents at C3 can determine the selectivity for a single galectin (Hsieh et al., 2016).

**Figure 6:**
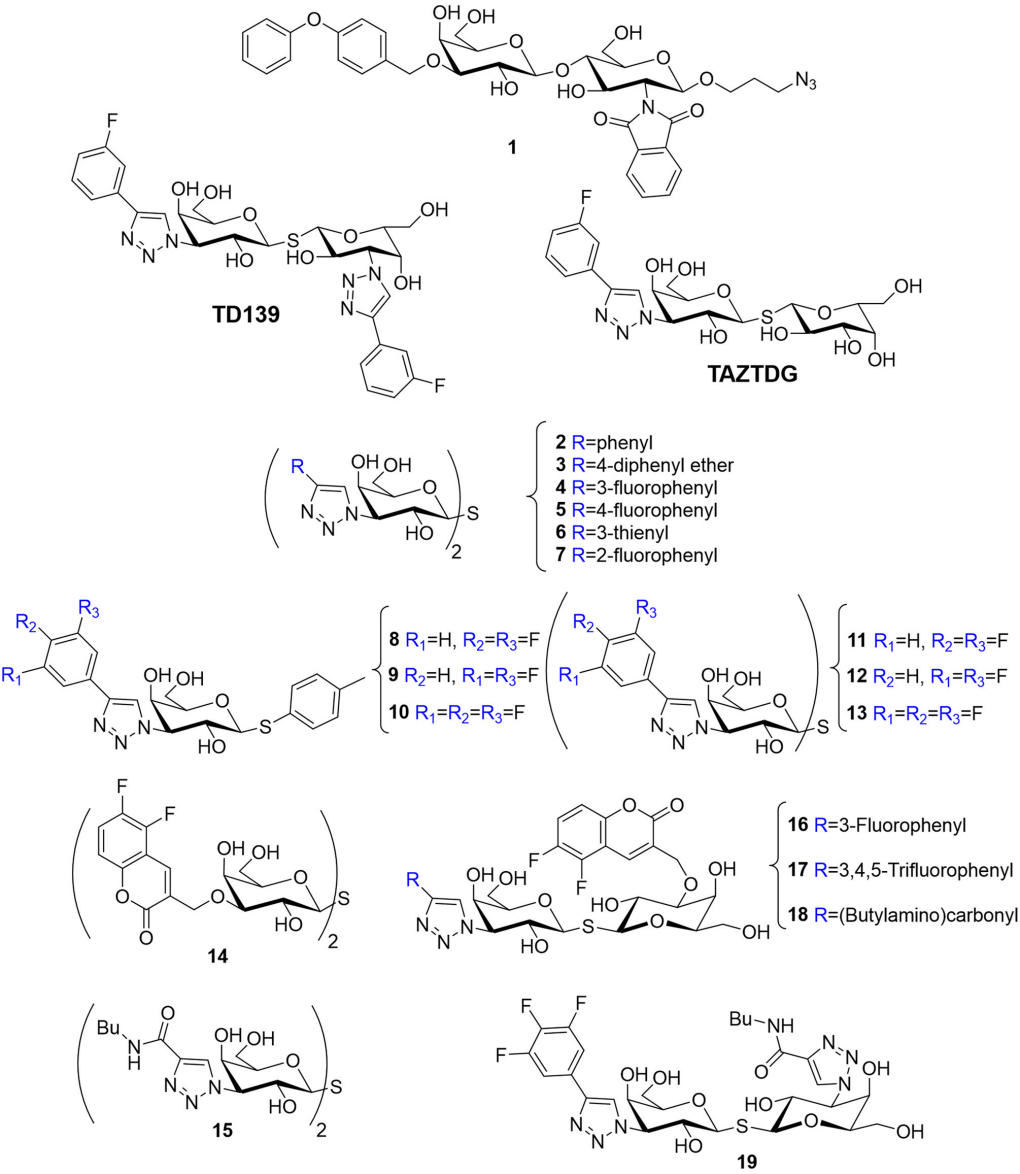
Structure of mimetics **1** (Van Hattum et al., 2013), **TAZTDG** (Hsieh et al., 2016), **TD139** (MacKinnon et al., 2012), **2**–**6** (Delaine et al., 2016), **8**–**13** (Peterson et al., 2018), **14** (Nilsson et al., 2013), **15** (Salameh et al., 2010), and **16**–**19** (Peterson et al., 2018).

Delaine et al. (2016) have presented the synthesis of a battery of doubly C-3 substituted derivatives at C3 (compounds **2-7**, Figure 6). The generated 4-aryl-1,2,3-triazolyl thiodigalactoside-based derivatives were tested as antagonists of galectin-1, -2, -3, -4 (*N*- and C-terminal domain), -7, -8 (N-terminal domain), domain), and 9 (N- and C-terminal domain) (Delaine et al., 2016). The molecules displayed affinities considerably larger than those measured for the unsubstituted thiodigalactosides, especially against *h*Gal-1 and *h*Gal-3. Regarding *h*Gal-1, this lectin bound the phenyl-triazole unsubstituted **2** with higher affinity (4 nM) than that for mimetics displaying larger substitutions (**3**, 84 μM). Furthermore, only the mimetic substituted with 3- or 4-fluorophenyl moieties (**4**, 12 nM; **5**, 27 nM, respectively) were more akin than unsubstituted **2**, strongly suggesting that the galectin subsites display rather limited possibilities for substitution, as previously described (Van Hattum et al., 2013). Nevertheless, the thienyl substituted compound **6** provided a very good inhibition of *h*Gal-1 (*K*_*D*_ < 10 nM, 2,400-fold stronger than TDG). Moreover, its selectivity for *h*Gal-1 over *h*Gal-3 (65 nM) was remarkable. Concerning *h*Gal-3, the selectivity profile was similar to that achieved for *h*Gal-1, with the exception of the 4-phenoxy-substituted phenyl **3** that displayed a *K*_*D*_ for *h*Gal-3 of 0.36 μM, with a considerably enhanced selectivity over *h*Gal-1 (*K*_*D*_ of 84 μM). It is evident that the right choice of the substituent on the triazole ring of this type of compounds guide selectivity for either *h*Gal-1 or *h*Gal-3.

Regarding *h*Gal-4, the aryltriazoles carrying no substituents or holding small appendages at the aryl moiety (**2**, **4**, **5**, **6, 7**) were the best inhibitors, especially for the C-terminal domain (mid-nM affinities), in line with the observations for *h*Gal-1. In contrast, the N-terminal domain recognized all the molecules of the study with low-medium μM affinities, with some notable exceptions (**4**, 0.17 μM). The best inhibitor for the C-terminal domain was the fluorophenyl-derivative **5** (73 nM). However, this molecule is not specific, since it also recognized *h*Gal-1 and *h*Gal-3 within the same affinity range. *h*Gal-7 and *h*Gal-9 (both C- and N-terminal domains) did not efficiently bind the larger compounds of the library, while they showed sub- to low-μM affinities for those carrying small substituents. In contrast with the data for *h*Gal-4, no binding preferences were detected when compared the C- and N-terminal domains of *h*Gal-9.

X-ray crystallography structures of *h*Gal-3 complexed with the three best antagonists (**4**, **5**, and **6**), together with mutation analysis of the lectin, showed that the aryl-triazol moieties display guanidine-arene interactions involving R144 and R186, as well as additional fluorine-amide orthogonal multipolar interactions, which are obviously absent in the binding with the natural ligands. The inhibition action of **4** against *h*Gal-3 was also corroborated with *in vitro* and *in vivo* strategies. The results showed that **4** has intracellular availability and activity blocking the intracellular accumulation of the lectin around damaged vesicles in breast carcinoma MCF-7 cells and also attenuates lung fibrosis in a dose-dependent manner, using a murine model (Delaine et al., 2016).

On the basis of this success, a systematic tuning of the aryl substituents was performed: a series of fluorinated C3 aryltriazolyl-substituted thiodigalactosides, including symmetrical and asymmetrical compounds, were synthesized to optimize the interaction with R144 of *h*Gal-3 (compounds **8-13**, Figure 6). As a first step, the interaction of monogalactosides carrying phenyl-triazole moieties with fluorine, chlorine, or bromine atoms was first evaluated, and the three best inhibitors in terms of both affinity and selectivity for *h*Gal-3 over *h*Gal-1 were selected. They include the di- or trifluorophenyltriazol thiogalactosides (**8**, **9**, and **10**), with relative affinities of 8.8, 15, and 5.2 μM, respectively. Then, the corresponding symmetrical thiodigalactosides (**11**, **12**, **13**) were prepared, which displayed improved affinity and selectivity compared to **4** and **5**, as well as higher affinities over the thiogalactosides **8**, **9**, and **10**. However, the selectivity was lower (Peterson et al., 2018).

In contrast, asymmetrically substituted thiodigalactosides showed improved binding affinities and selectivity. The design of these new compounds was based on the good selectivity described for the analogous symmetrical molecules **14** (Nilsson et al., 2013) and **15** (Salameh et al., 2010) and they combined the monofluoro- and the trifluorophenyl moieties present in **4** and **13** with the coumaryl and triazolyl groups in **14** and **15** (Figure 6). This new set of compounds (**16**, **17**, **18**, and **19**) improved *h*Gal-3 over *h*Gal-1 selectivity maintaining the low nM affinity (Figure 6). In particular, **17** (7.5 nM) was 46-fold more affine for *h*Gal-3. Structural analysis and ITC measurements revealed that increased fluorination of a phenyl triazole group favors the interaction with R144. In conclusion, the combination of C3-aryltriazolyl groups with O3-coumaryl in a thiodigalactoside generates a first-class glycomimetic with affinity and selectivity for *h*Gal-3 without equals (Peterson et al., 2018).

**Figure 7:**
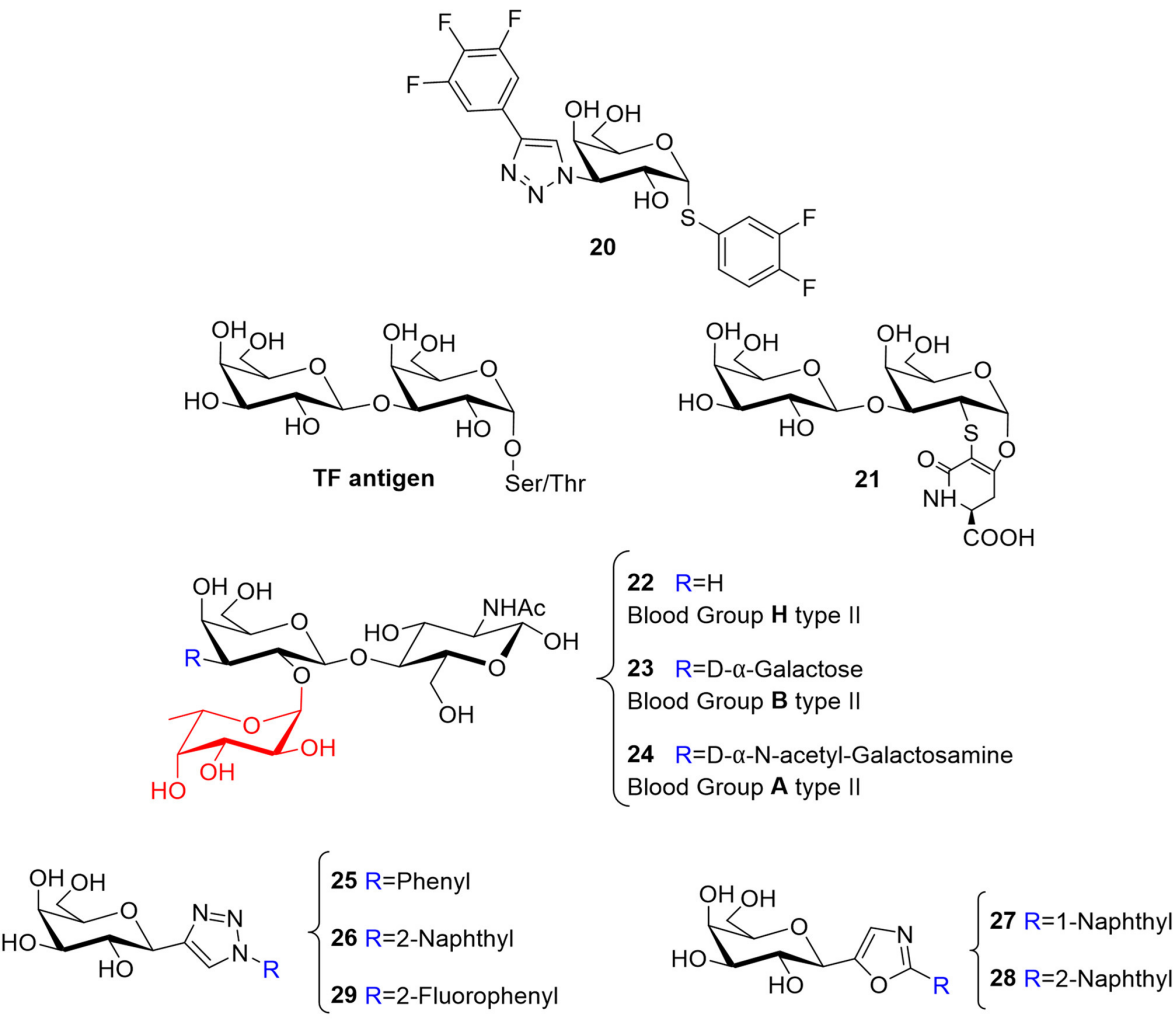
Structure of mimetics **20** (Zetterberg et al., 2018), **21** (Santarsia et al., 2018), and **25**–**29** (Dahlqvist et al., 2019). Structure of TF antigen and histo-blood group antigens H, B, and A of type II (**22–24)**.

Additional improvements based on the monogalactoside scaffolds **8**, **9**, and **10** have also been achieved. Zetterberg et al. (2018) used a different approach, derivatizing the Gal moiety at position 1 with an unnatural substituent, thus favoring non-natural lectin-ligand interactions. They include orthogonal multipolar fluorine-amide, phenyl-arginine, sulfur-π, and halogen bond interactions. All the resulting compounds [series of α- and β-thio-D-galactopyranosides with 4-(3,4,5-trifluorophenyl)-1H-triazole at C3] bind to *h*Gal-3 with very good affinities. In particular, the S-glycoside **20**, substituted with a 3,4-dichlorophenylthio moiety (Figure 7) presents a *K*_*D*_ of 37 nM, and it is fairly selective, with 100-fold lower affinity for most of the other galectins (1, 4N, 7, 8C, 8N, 9C, 9N) and 20- and 4-fold for *h*Gal-2 and *h*Gal-4 C-terms, respectively (Zetterberg et al., 2018).

The cellular uptake of some of these mimetics (**4** with *K*_*D*_ of 12 nM, **20** with *K*_*D*_ of 37 nM, and **15** with *K*_*D*_ of 99 nM) and their *h*Gal-3 inhibition ability have been evaluated in intracellular and extracellular environments (Stegmayr et al., 2019). The permeability assays with monolayers of Caco-2 cells determined that **20** had a significantly higher value of membrane permeability (with passive transport) than **4** and **15**. Despite the relatively similar affinities, their polar surface area is different, being significantly lower for **20**, in good correlation with the observed results. The extent of non-polar surface areas is relevant for the passive cell membrane permeability and oral bioavailability. Additionally, the extracellular activity was tested using an assay based on the use of a fluorescently-labeled *h*Gal-3 coupled to the cell surface of CHO cells line, revealing that the inhibition potency correlated with the previously measured relative affinities. The determination of the intracellular inhibition was based on the induction of the accumulation of *h*Gal-3 around damaged intracellular vesicles, followed by the subsequent administration of the inhibitors. The results followed the trend of the permeability assay, with **20** showing IC50 values considerably lower than those for **4** and **15** (Stegmayr et al., 2019).

Another promising disaccharide mimetic has been presented by Marcelo and co-workers. The authors focused on the interplay between *h*Gal-3 and the Thomsen-Friedenreich (TF) antigen to develop a mimetic capable of binding *h*Gal-3 and compete with the natural antigen (Santarsia et al., 2018). The TF antigen (**20**), also known as pancarcinoma-related antigen, is a disaccharide (Gal**β**1 → 3GalNAc) that decorates glycoproteins located on the cell-surface through O-Ser/Thr linkages (Figure 7). Under healthy conditions, this fragment is not exposed since it is covered by additional glycosylation, and therefore hidden. In contrast, the epitope is exposed and overexpressed in the tumor microenvironment, particularly in adenocarcinomas (Springer, 1997; Glinsky et al., 2001). Now, the TF antigen is accessible and, among other interactors, extracellular *h*Gal-3, also overexpressed in various tumor lines, is capable of recognizing and binding to it, so mediating the adhesion of tumor cells to the endothelium leading to metastasis progression (Jeschke et al., 2006; Fortuna-Costa et al., 2014; Mori et al., 2015). MUC1 is a tumor-related glycoprotein decorated with the TF antigen, and it has been proposed that the aberrant interaction between MUC1 presenting TF disaccharide epitopes, and *h*Gal-3 promotes cancer progression (Glinsky et al., 2001; Goletz et al., 2002; Takenaka et al., 2002). The authors exploited a previously proposed α-O-Gal scaffold (Jiménez-Barbero et al., 2009) to generate the disaccharide TF mimetic (**21**, Figure 7). ^15^N-^1^H HSQC-based titrations provided the chemical shift perturbations of the *h*Gal-3 signals, which revealed that the more affected protein residues belong to the canonical binding site already described for the natural TF antigen, both by NMR and X-ray crystallography (Bian et al., 2011; Yongye et al., 2012). Linewidth ^1^H NMR and STD-NMR experiments allowed to obtain the ligand-binding epitope (Valverde et al., 2019b; Gimeno et al., 2020). Both experimental procedures pointed out that the disaccharidic moiety is the key epitope for *h*Gal-3, while the unnatural scaffold is exposed to the solvent. Similar observations have been reported for the natural ligand TF, where the attached Thr does not participate in the binding event (Santarsia et al., 2018). The thermodynamic profile of the system indicate that there is a slight gain in terms of *K*_*D*_, which interestingly arises from a favorable entropic contribution (−0.9 kcal mol^−1^), while there is less enthalpy gain (−4.3 kcal mol^−1^) compared to the natural TF (–TΔS 7.75 kcal mol^−1^; ΔH − 12.6 kcal mol^−1^) (Yongye et al., 2012). The analysis of the X-ray crystallographic structure obtained for the *h*Gal-3/**21** complex allowed noticing the absence of four hydrogen bonds involving the GalNAc residue that were present in the complex with the TF antigen, due to the chemical nature of the lactam ring and the removal of the NHAc group. These evidences might explain the loss of enthalpy gain; however, they did not provide a completely definite explanation for the observed positive entropic term. The authors suggested that the amphiphilic character of the lactam ring and the associated changes in the solvation features could be beyond the favorable entropic contribution. This work is an exquisite example of how a simple modification of the chemical nature of the ligand can lead to a big change in the entropy-enthalpy compensation phenomenon without changing the binding epitope. Overall, mimetic **21** might be considered as a putative lead compound to interfere with the aberrant interaction of *h*Gal-3 and the TF antigen (Santarsia et al., 2018).

In this context, and concerning the role of the entropy, a multidisciplinary study of the interaction between the histo-blood group antigens (Figure 7, *h*BGAs type II **22**, **23**, **24**) and *h*Gal-3, revealed that the typical entropy penalty that occurs upon galectin binding may be modulated by chemical modifications at the ligand. For the *h*BGA tetrasaccharides, which are fucosylated molecules, it was demonstrated that the fucose moiety rigidifies the motion around the glycosidic linkages of the whole molecule, thus providing a rather stiff and preorganized conformation. This feature is reflected in the existence of faster association rates of these ligands to *h*Gal-3, which also exhibit a less unfavorable binding entropy. Even if the fucose is not directly involved in the binding event, it is capable of guiding the interaction and improving the binding affinity. This evidence may be considered to explore novel avenues for the development of more potent ligands (Gimeno et al., 2019).

Simple *C*-glycosyl compounds have also been employed as scaffolds for developing selective small-molecule inhibitors, mainly involving C1- and C3-substitutions at the Gal moiety. Nilsson and collaborators have proposed novel C1-galactopyranosyl heterocycle mimetics that were fine-tuned against galectins (Dahlqvist et al., 2019). Different triazoles, oxazoles, isoxazoles, and pyrazoles (**25–29**, Figure 7) were used as substituents at Gal C1 to generate a library that was tested with galectins 1, 3, 4, 7, 8 (C- and N-terminal), and 9 (C- and N-terminal). Competitive fluorescence polarization assays (Sörme et al., 2004) allowed discriminating the more active compounds. The triazole-substituted **25** and **26** mimetics were fairly selective for *h*Gal-1 (*K*_*D*_ 750 μM and 290 μM, respectively) while the oxazole-substituted **27** and **28** were selective for *h*Gal-3 (*K*_*D*_ 90 μM and 230 μM, respectively). These compounds presented affinities only in the millimolar range with all the other galectins. On the other hand, the isoxazoles displayed good affinity but poor selectivity, while the pyrazoles were poorly active and selective. Consequently, further design was accomplished using the triazoles and the oxazoles as leads. Better affinity for *h*Gal-1 was found for triazole **29** (*K*_*D*_ 170 μM), where the aryl moiety is fluorinated at position 2, keeping the specificity for this galectin. Molecular Dynamics (MD) simulations performed for the *h*Gal-1/**29** complex revealed a stacking interaction between the triazole ring of **29** and the His52 side chain of *h*Gal-1. This His52 is unique for *h*Gal-1 and differentiates it from the other galectins. The specificity of **27** for *h*Gal-3 was accounted for by the occupation of the ligand of a unique subsite of the lectin above the β-face of the bound Gal ring. The aforementioned workflow can be followed to develop more high-affinity inhibitors by exploiting the reported heteroaryl-selectivity (Dahlqvist et al., 2019).

Besides sugar-based mimetics, non-carbohydrate inhibitors have also been studied. In particular, carbohydrate-mimetic peptides have been developed as effective inhibitors, taking advantage of the role that peptides may play as immunogens and interfere with protein–sugar interactions (Kieber-Emmons et al., 2014). Using high-throughput phage display analysis, a variety of synthetic peptides capable of binding to the CRD of *h*Gal-3 with high affinity, were identified as well as their inhibition ability to affect metastasis-associated cancer cell adhesion (Zou et al., 2005). The most active peptides shared the common motif [P-(C)-G-P-X-X-X-D-(C)-P]. Notably, **G3-C12** (ANTPCGPYTHDCPVKR) recognized *h*Gal-3 with a *K*_*D*_ of 70 nM, and its administration significantly reduced metastatic cell deposition in a mice model, consequently decreasing the outgrowth within vasculature (Newton-Northup et al., 2013). Using N-(2-hydroxypropyl)methacrylamide (HPMA) as a carrier molecule, the drug delivery process was further enhanced (Yang et al., 2014).

**Figure 8:**
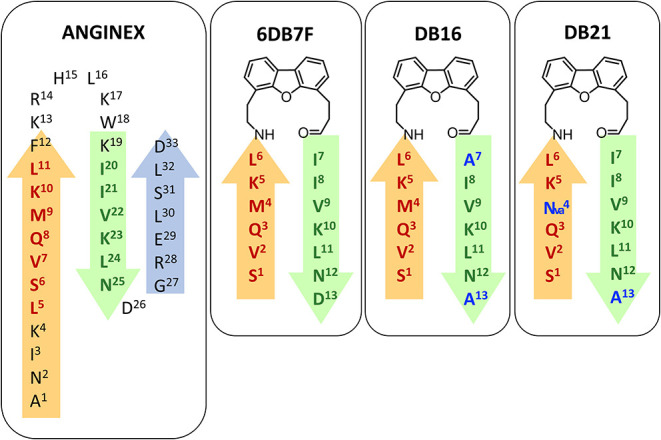
Amino acidic sequence of different mimetics: **anginex** (Mayo et al., 2001; Griffioen, 2006), **6DB7F** (Mayo et al., 2003, 2008), **DB16**, and **DB21**. β-sheets structures are reported as arrows.

**Anginex** is an amphipathic synthetic peptide directed against *h*Gal-1, designed through basic folding principles, which incorporates short sequences of interleukin-8, platelet factor-4 and the bactericidal/permeability increasing protein (Figure 8), (Mayo et al., 2001; Griffioen, 2006). It is able to act as tumor growth inhibitor and to block angiogenesis and migration of cancer cells (Griffioen et al., 2001; Wang et al., 2012). It adopts a major β-sheet topology, as deduced by NMR and CD experiments, and displays a net positive charge as well as a hydrophobic face that are essential for its function (Dings et al., 2003; Mayo et al., 2003). Concerning the specificity, anginex preferentially targets *h*Gal-1, although it is also capable of binding to *h*Gal-2, -7, -8N, and -9N with low affinities. In contrast, it does not show any activity against *h*Gal-3, -4N, -4C, and 9-C 9-C (Salomonsson et al., 2011). Starting from **anginex**'s sequence, other antagonist peptides of *h*Gal-1 with improved properties have been developed. Mayo et al. (2003, 2008) synthetized **6DBF7**, in which keeping the β-sheet structure presents a central dibenzofuran (DBF) scaffold that connects six amino acid residues (at the N-term) to other seven amino acids (at the C-term) (Figure 8). **6DBF7**, which also targets *h*Gal-1, is more effective *in vivo* in terms of anti-angiogenic and anti-cancer activity than parent **anginex** (Mayo et al., 2003, 2008). A soluble analog, **DB16**, was employed to deduce the binding site by ^15^N-^1^H HSQC NMR experiments. The interaction primarily takes place at one edge of the β-sandwich of the lectin and reduces the affinity of *h*Gal-1 to lactose, suggesting the presence of an allosteric interplay (Dings et al., 2013). The **DB21** analog, which shows a branched alkyl side chain (Figure 8) shows even higher capacity to inhibit tumor angiogenesis and tumor growth *in vivo*.

**Figure 9:**
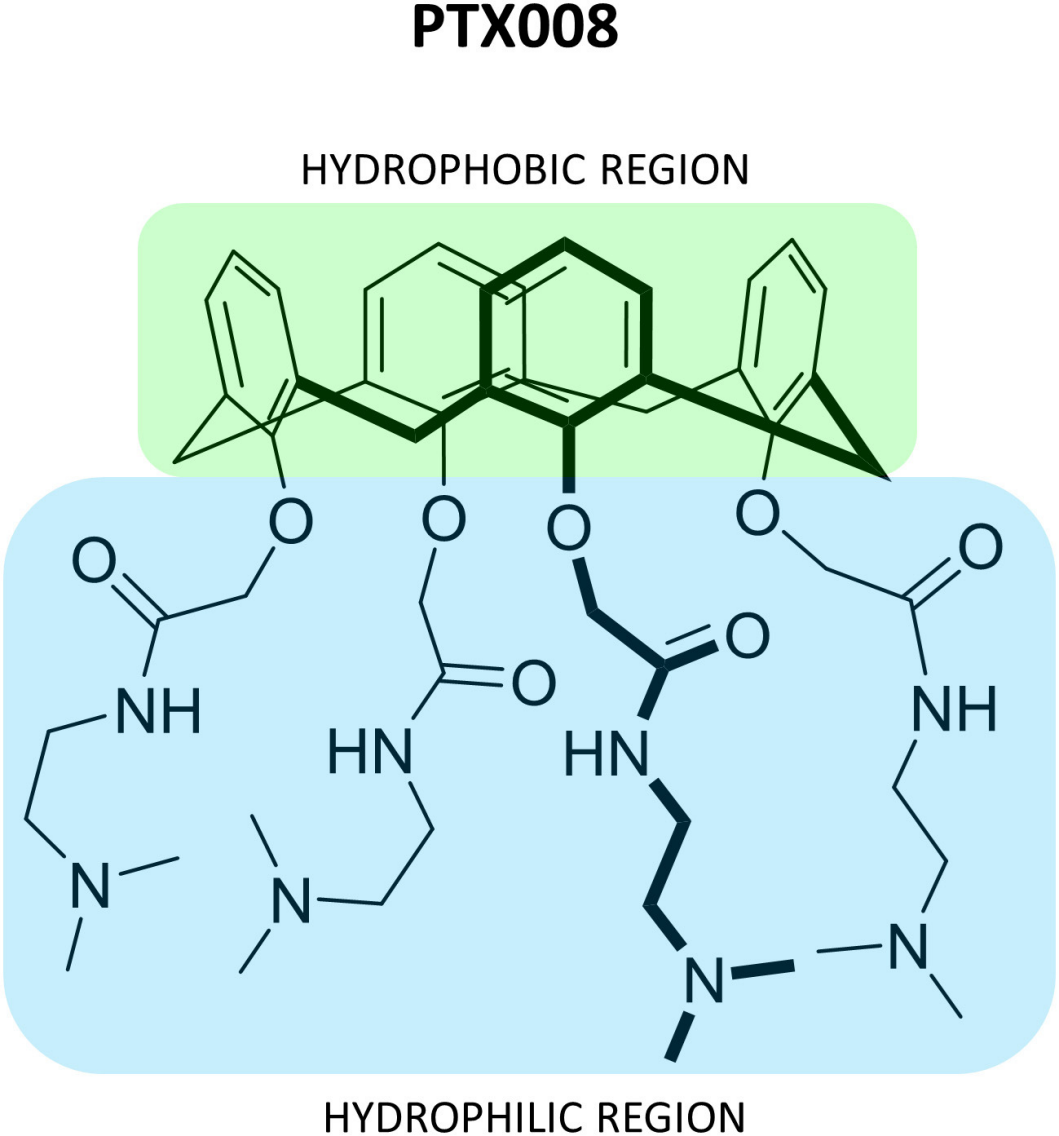
Representation of compound **PTX008** (Dings et al., 2006; Mayo, 2014).

A series of non-peptidic topomimetics of anginex based on a calix[4]arene scaffold substituted with hydrophobic and hydrophilic moieties have also been employed (Dings et al., 2006). Compound **PTX008** (also known as OTX008) (Figure 9) interacts with the lectin at an allosteric site (Dings et al., 2008) and shows potent anti-angiogenic and anti-proliferation activity *in vitro* and *in vivo*. In fact, it was approved for Phase I clinical trials for advanced solid tumors (NCT number: NCT01724320) (Thijssen et al., 2006; Mayo, 2014; Koonce et al., 2017).

A series of peptides and phosphopeptides able to interact with the *h*Gal-3 CRD were also identified using fragments from the N-terminal tail of *h*Gal-3. HSQC experiments using ^15^N-labeled *h*Gal-3 CRD demonstrated the effective interaction of the peptides with the back-face of the CRD of *h*Gal-3, also showing that phosphorylation of serines 6 and 12 enhances the binding, while phosphorylation of tyrosines 107 and 118 in specific N-peptides attenuates the interaction. These data also suggested a fine modulation of *h*Gal-3 activity through the phosphorylation of the tail (Berbís et al., 2014).

## Multivalent Presentation Strategies

In biological systems, low-affinity binding is usually overcome through the engagement of simultaneous interactions that the receptor and ligand can establish a phenomenon known as multivalency (Chittasupho, 2012). Thus, mimicking nature and developing multivalent ligands has been a frequent approach to surmount the low affinity of single galectin-carbohydrate interactions.

There are diverse ways in which multivalent presentations can enhance affinity: chelation, subsite binding, statistical rebinding, steric stabilization, and clustering effects (Cecioni et al., 2015; Zhang et al., 2018; Bücher et al., 2019). However, in the development of multivalent ligands, there are various factors that should be taken into consideration. First, the nature of the scaffold. Rigidity is a crucial feature to consider, since it is directly related to entropy. Flexible linkers may display a large entropic penalty upon binding. Second, flexibility can also be advantageous because the linker might adopt the proper conformation for favorable interactions to take place (Shewmake et al., 2008; Hevey, 2019). Third, the chemical nature of the linker is also relevant, since it might establish additional interactions with secondary binding sites and therefore improve the affinity (Boden et al., 2019). Undoubtedly, the choice of the ligand is a key factor in the outcome as well as its actual concentration within the scaffold. Usually, the higher the concentration of the ligand is, the higher the affinity. However, at a high concentration of the ligand, steric clashes may take place and the effectivity of the approach decreases. Thus, the content of the specific ligand should be carefully chosen (Karaman, 2010).

**Figure 10:**
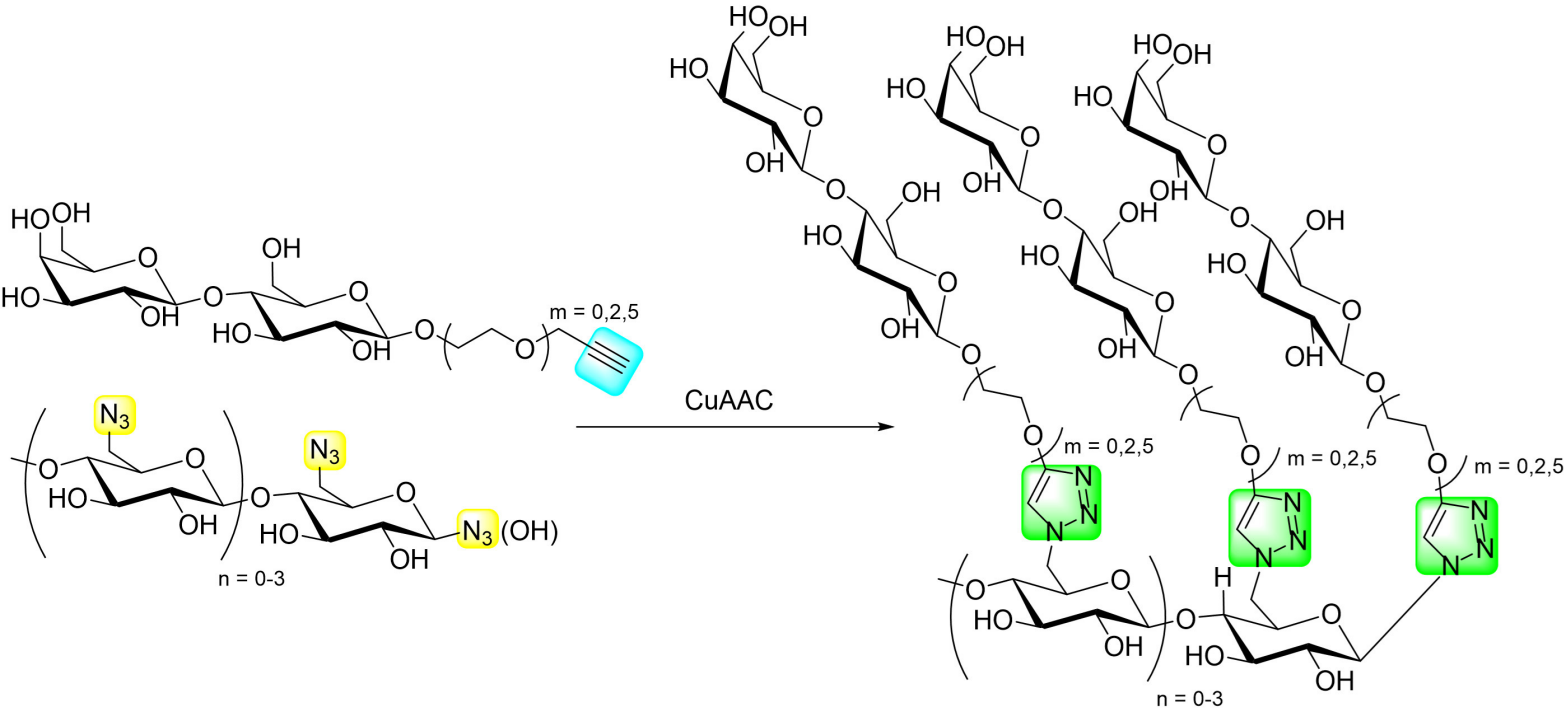
The multimeric lactosides employed by Gouin et al. (2010).

Despite that most of the described antagonists targeting galectins are monovalent compounds, diverse multivalent glycomimetics have been developed over the last decade. One of the first designs of multivalent ligands targeting galectins was proposed by Gouin et al. (2010). Multivalent ligands with a glucose-based backbone and a lactose epitope targeting *h*Gal-1 and *h*Gal-3 were designed (Gouin et al., 2010; Figure 10). In order to address the relevance of valence, different multimeric compounds were synthesized, varying the number of glucose units and therefore the number of lactose moieties available in the construct. The pendant chains also present ethylene glycol spacers with different lengths, which affect the 3D structure and flexibility of the cluster. Moreover, they were attached to the β-1,4-linked glucose backbone using positions 1 or 6, thus, leading to a variety of compounds that enabled the independent analysis of the effect of epitope-units and the spacer length (Rostovtsev et al., 2002; **Figure 13**). This strategy has previously been applied for the synthesis and binding studies of multivalent mannosides with ConA (Gouin et al., 2007).

Competitive fluorescence polarization assays demonstrated that the spacer length of the multimeric lactosides has no relevant effect, since negligible differences in affinity toward *h*Gal-1 and *h*Gal-3 were observed (Rostovtsev et al., 2002). In fact, MD calculations suggested that the lactoside moieties display a random localization, and the spacer length does not alter the availability of the binding epitope. Two-site ELLA experiments were employed to test the crosslinking ability of the multilactosides, first with peanut agglutinin. In this case, the relative potency per lactoside ranged from 2.1 to 3.2 (McCoy et al., 1984). However, the crosslinking ability was not reproduced for galectins, for which the affinity increased in proportion to the lactose content. The best glycomimetic was the trivalent compound, which yielded a *K*_*D*_ of 16 μM toward *h*Gal-3, a <5-fold affinity increase in comparison with the monovalent ligand.

A different glucose-based scaffold to bear disaccharide epitopes has been proposed by Rahkila et al. (2019), consisting of a dextran skeleton decorated with LacNAc moieties. The idea behind this architecture was to mimic natural polysaccharides (Rahkila et al., 2018). In particular, the multivalent antagonists used a ca. 70 kDa dextran, which was propargylated and bioconjugated with lactose, as well as with mannobiose and maltose disaccharides (Grischenko et al., 2013) as blank molecules. Interestingly, upon disaccharide coupling through CuAAC, fractionation of the dextran occurred, yielding multivalent ligands of ca. 40–50 kDa. Binding studies of the multivalent conjugates were performed through ^15^N-^1^H HSQC NMR titrations with the carbohydrate recognition domain of *h*Gal-3 and dextrans decorated with different lactose contents were evaluated. Surprisingly, dissociation constants ranging from 0.25 to 0.45 mM were recorded, very similar to those reported for free lactose (Diehl et al., 2010; Miller et al., 2016) The lack of affinity enhancement suggests that the multiple interactions do not cooperatively provide additional value to the interaction, which might arise from unfavorable entropic costs (Bernardi et al., 2014). Strikingly, although the titration with the mannobiose multivalent ligand produced small chemical-shift perturbations, a considerable decrease in the signal intensities was observed in ca. 15 peaks, not located at the canonical binding site of the lectin. Interestingly, these residues are located in the opposite face of the binding site of the lectin and belong to the region that was previously reported to interact with β-mannans (Miller et al., 2016). Although the obtained affinities did not provide positive multivalence effects, probably due to the entropic costs caused by the flexible linker, and the dextran backbone constitutes an easy to modify, well-defined, and homogeneous scaffold to mimic natural-like polysaccharides with possible applications as biomaterials.

**Figure 11:**
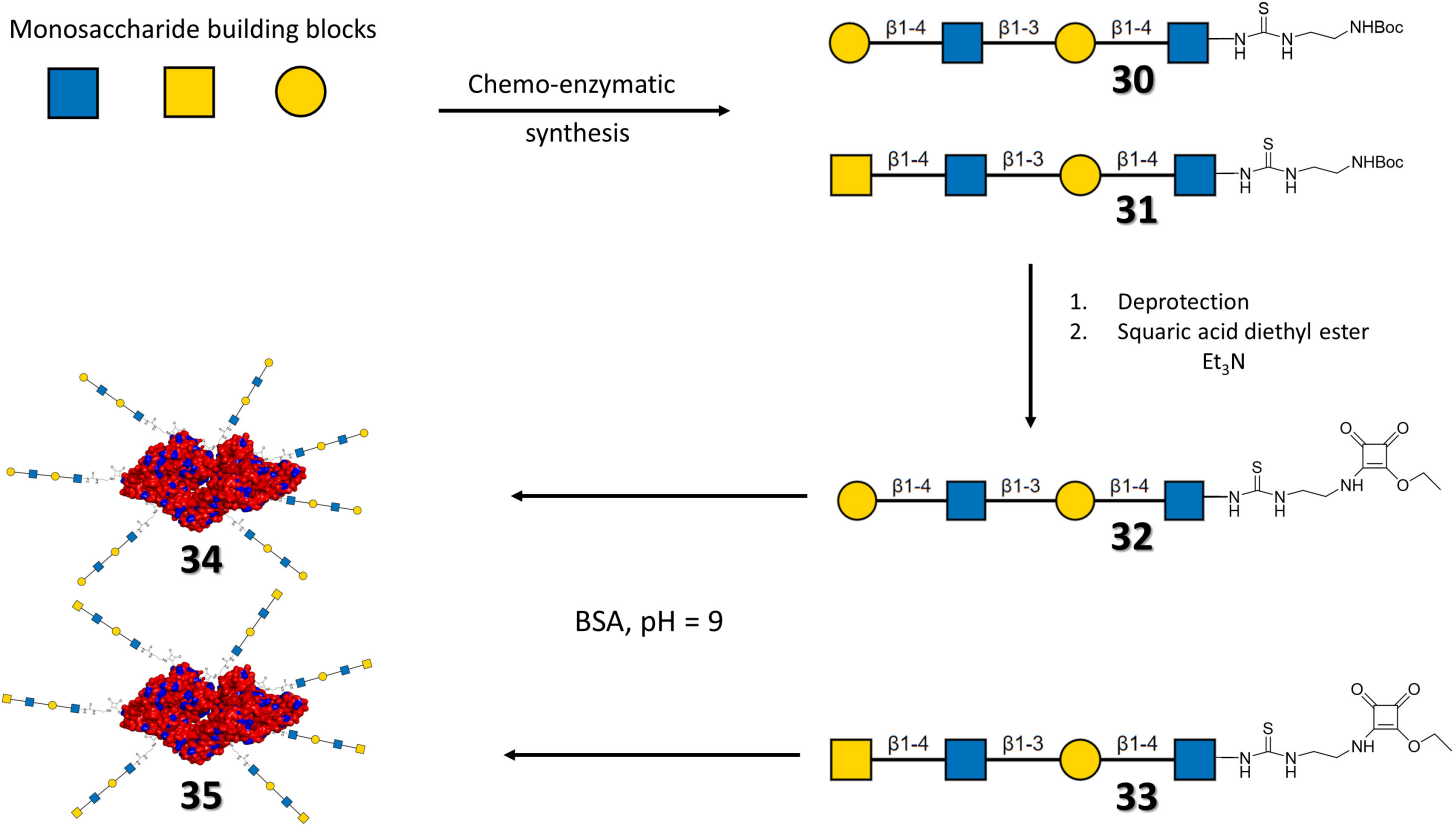
General scheme of the synthesis of the neo-glycoproteins. For neo-glycoproteins series 35 and 36 the surface of BSA is depicted in red, except for lysine residues (blue), the attached carbohydrates are drawn with their symbolic representation. For each series of neo-glycoprotein, the number of lysines glycosylated ranged from 0 to 29.

Alternatively, Böcker et al. (2015) have employed neo-glycoproteins based on bovine serum albumin (BSA) to target human *h*Gal-3. BSA is a highly abundant protein rich in lysine residues, which makes it fairly appropriate for bioconjugation (Blackmore and Eisoldt, 1999; Huang et al., 2004; Luyai et al., 2009). Two different tetratrasaccharides **(30, 31)** were synthesized in a chemo-enzymatically manner and coupled to squaric acid diethyl ester to provide **32** and **33**, which were conjugated to BSA to provide two neo-glycoproteins series **(34, 35)** with specific glycans attached at different (between 0 and 29) lysine residues (Figure 11).

The selective binding of the multivalent compounds to *h*Gal-1 and *h*Gal-3 was tested through ELISA-type assays (Kupper et al., 2013). The BSA conjugated with the LacNAc-LacNAc motif (**34**) displayed preference for *h*Gal-3, as a result of the capability of this lectin of recognizing the internal LacNAc motif, which is rather weak for *h*Gal-1 (Stowell et al., 2004). Interestingly, the selectivity for *h*Gal-3 decreased when the carbohydrate content increases, even if the affinity was better. For BSA conjugated with the LacDiNAc-LacNAc tetrasaccharide (**35**), the affinity also increased with a higher carbohydrate content. However, the selectivity between the lectins decreased. The selectivity of ligands **35** toward *h*Gal-3 was always higher, given the low capability of *h*Gal-1 to recognize LacDiNAc moieties (Van den Berg et al., 2004; Rapoport et al., 2008). Therefore, the interaction relies on the weak recognition of the internal LacNAc. The affinity increase per glycan unit was then analyzed at different lectin concentrations. The LacNAc-LacNAc series **34** did not show significant affinity enhancement. In contrast, the LacDiNAc analogs **35**, with 14 to 27 glycosylated lysines, displayed multivalent effects with a 100-fold increase in the relative potency per glycan. Overall, the best neo-glycoproteins showed very high affinity (in the nM range) and selectivity toward *h*Gal-3.

As previously described, the addition of hydrophobic residues to the Gal moieties at proper positions enhances the affinity toward galectins. However, the corresponding synthetic approach may involve laborious chemical procedures (Sörme et al., 2003). In order to overcome this issue, Böcker and Elling (2017) biotinylated the glycan epitopes attached to BSA at position 6 of the non-reducing end through enzymatic synthesis (Böcker and Elling, 2017). Fittingly, the presence of biotin increased the affinity of the ligands toward *h*Gal-1 and *h*Gal-3, maintaining the selectivity for *h*Gal-3. In fact, comparing biotinylated and non-biotinylated neo-glycoproteins, the latter required higher glycosylation densities to reach similar multivalent effects. In the presence of biotin, six glycosylations were enough to obtain a *K*_*D*_ of 100 nM, whereas, in the absence of biotinylation, only the analog with 14 glycosylations displayed a similar binding affinity.

**Figure 12:**
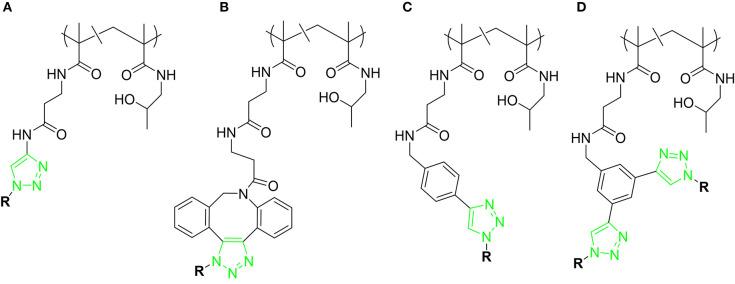
Different scaffolds **(A–D)** used for targeting *h*Gal-3. *R* = LacDiNAc. For each scaffold, polymers with different carbohydrate content were synthesized.

The LacDiNAc epitope has been widely employed for decorating scaffolds in the seeking of selectivity. Bojarová et al. (2018) have selectively targeted *h*Gal-3 by employing a series of HPMA [N-2(2-hydroxypropyl)methacrylamide]-based nanocarriers (Figure 12) bearing this disaccharide motif (Bojarová et al., 2018). HPMA, a biocompatible nanocarrier, soluble in water and non-toxic (Ulbrich and Šubr, 2010), was conveniently functionalized with alkyne and easily conjugated to the desired sugar epitope. The neighborhood and content of the alkyne-bearing groups was varied to provide multimeric ligands with different carbohydrate contents (3 to 29 mol-%), providing a platform to evaluate structure-activity relationships (Figure 12). Additionally, the effect of the presence of a short linker between the disaccharide and the triazole moieties was also investigated.

Competitive inhibition ELISA-based assays of *h*Gal-3 binding to asialofetuin determined that a sugar content between 8 and 12 mol-% was optimal for providing the best relative potency (r_p_/n) compared to the single LacDiNAc disaccharide. Direct attachment of the sugar to the triazole moiety resulted in an improvement in affinity compared with the more flexible O-linked 2-ethylazido linker. Additional contacts between the electron-rich ring with Arg186 at subsite E of *h*Gal-3 are probably at the origin of this effect. The presence of a phenyl group attached to the triazole moiety (scaffold C) did not improve the affinity when compared to scaffold A, whereas the presence of the large DBCO linker (scaffold B) was detrimental for binding. Overall, the best results were obtained for scaffold A, with the LacDiNAc disaccharide (8.1 mol-% content) directly attached to the triazole moiety, although a small r_p_/n value of 2.3 was achieved.

**Figure 13:**
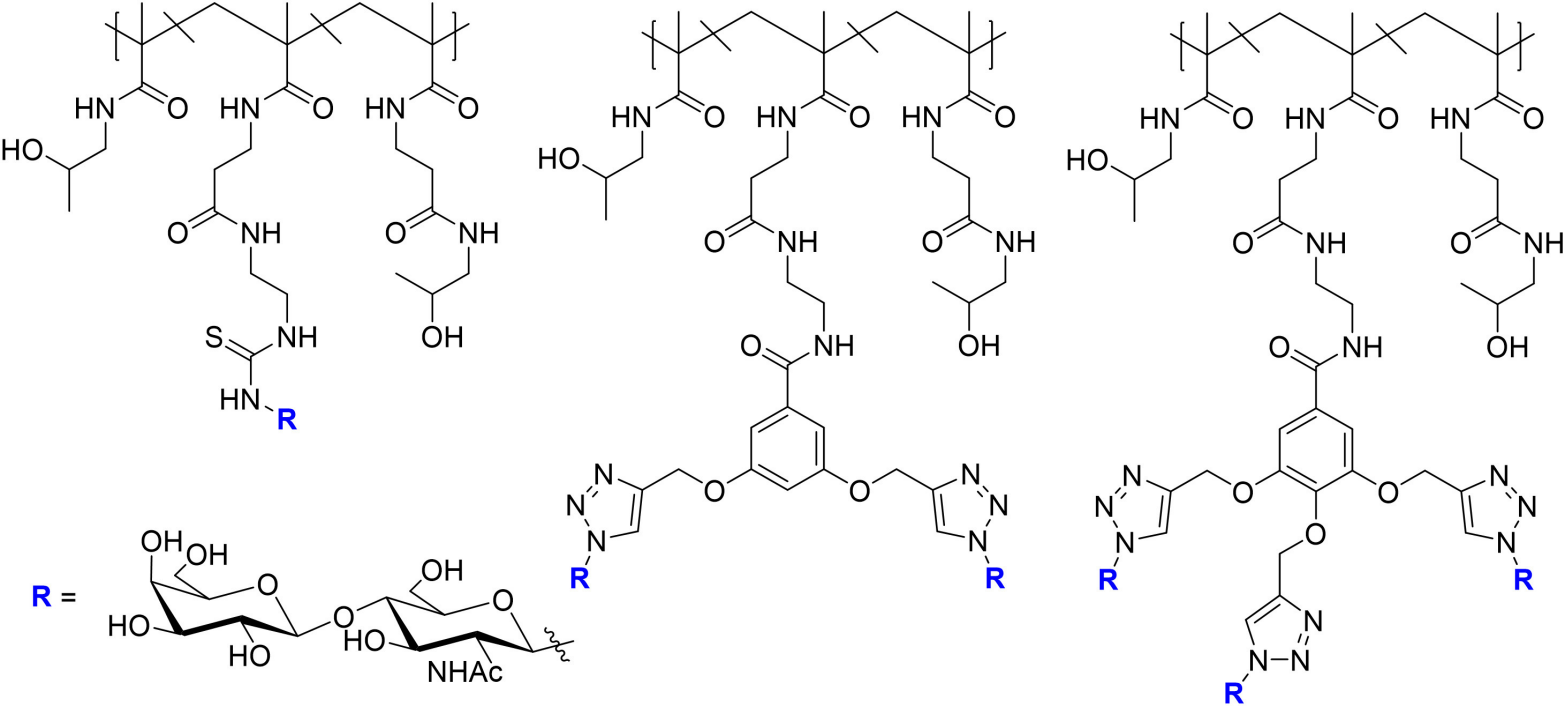
Glycopolymers designed for targeting *h*Gal-1 and *h*Gal-3. For each scaffold, five polymers were synthesized with different carbohydrate contents.

A series of HPMA-based glycopolymers (Figure 13) bearing different LacNAc contents (3.8 to 22.0 in mol-%) have been designed (Tavares et al., 2020) and tested through an ELISA-type assay.

The selectivity of the designed multimeric compounds for *h*Gal-1 was remarkable. The ligands displayed IC_50_ values in the μM range vs. *h*Gal-3, whereas nM affinities were achieved for *h*Gal-1. The best results were obtained for the glycopolymers carrying monovalent scaffolds, with 19.9 and 20.6 mol-% of glycan content, which displayed IC_50_ values in the low nM scale, and for which each LacNAc unit was 166 times more potent than that in the single presentation and 283 times more selective for *h*Gal-1 than for *h*Gal-3.

**Figure 14:**
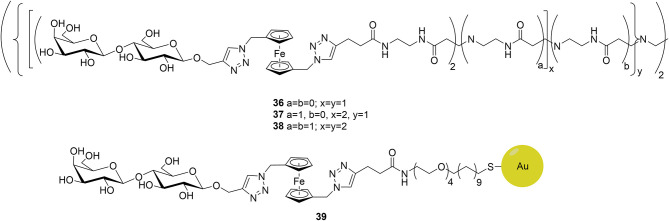
The lactose-ferrocene conjugates targeting *h*Gal-3.

In the lectin detection side, Martos-Maldonado et al. (2020) have developed a series of lactose-ferrocene conjugates to improve the sensitivity and reproducibility of *h*Gal-3 detection. This design was based in initial works that employed ferrocene-sugar dendrimers and improved the sensing ability toward a given lectin by combining the multivalent presentation features with the multielectron exchange capability due to the presence of multiple ferrocene units (Martos-Maldonado et al., 2013a,b). In particular, four different ligands were designed targeting *h*Gal-3, three dendrimers with different lactose content: **36** (4 lactose units), **37** (8), and **38** (16) and a monovalent ligand attached to gold nanoparticles, with more than 1,500 ligands per nanoparticle (Figure 14).

ITC data demonstrated that the affinity was higher for the multivalent dendrimers and improved for that with the highest valency values (**38**). Intriguingly, although the gold nanoparticle analog **39** displayed smaller affinity toward *h*Gal-3, its sensing capability was extraordinary, obtaining a detection limit of 160 nM. Overall, the three dendrimers displayed affinity values in the range of the most potent inhibitors previously developed, whereas the gold nanoparticle is a promising electrochemical probe that may enable the detection of *h*Gal-3 at biologically relevant concentrations.

## Conclusions

High affinity and selective antagonists have been designed, especially for *h*Gal-1 and *h*Gal-3, including various ligands that are in clinical trials, although none of them are in the market yet. The success of the mimetics designed on the basis of single presentation strategies relies on the fact that not only the canonical binding site of galectins has been targeted, but also interactions with neighboring regions have been exploited through the wise decoration of the sugar scaffold with specifically designed functional groups, using a canonical medicinal chemistry approach based on the continuous improvement of the binding enthalpy factor. The use of symmetric molecules, such as the TD139 and its pseudo-symmetric relatives, have demonstrated to be in the good pathway. Nevertheless, the control of the entropy factor, with the concomitant enthalpy-entropy compensation phenomenon, remains challenging and the next frontier, not only for the single presentation strategies but especially for the design of glycoconjugates with multiple epitope presentations. Except the chimera-type *h*Gal-3, the other galectins are dimers whose sugar-binding sites are located at opposite spatial directions. Therefore, there is no obvious strategy for tackling both binding sites in a simultaneous manner. The proposed scheme for the best molecule should keep enough flexibility to reach the target binding sites and thus enhance avidity, but without paying a high entropic penalty. Usually, typical lactose or N-acetyllactosamine molecules have been used to decorate the multivalent scaffold, what implies that single-binding events are of moderate affinity. There is room for improvement at this point, using specific functional groups and probably also using strategies that minimize the entropy loss at the nanocarrier and/or at the interaction site. The approaches employed so far have proved to be satisfactory and are good starting points for further efforts. New biological roles for galectins are continuously being shown. Thus, the design, synthesis, and applications of galectin binders and sensors will continue being an active field of research for the years to come.

## Author Contributions

AA and JJ-B developed the idea. SB, JQ, and AG wrote the review. AA, AG, and JJ-B revised the manuscript and gave it the final form. All authors contributed to the article and approved the submitted version.

## Conflict of Interest

The authors declare that the research was conducted in the absence of any commercial or financial relationships that could be construed as a potential conflict of interest.
